# A Proof-of-Concept Fragment Screening of a Hit-Validated 96-Compounds Library against Human Carbonic Anhydrase II

**DOI:** 10.3390/biom10040518

**Published:** 2020-03-29

**Authors:** Steffen Glöckner, Andreas Heine, Gerhard Klebe

**Affiliations:** Institute for Pharmaceutical Chemistry, Philipps-Universität Marburg, Marbacher Weg 6, 35037 Marburg, Germany; steffen.gloeckner@staff.uni-marburg.de (S.G.); andreas.heine@staff.uni-marburg.de (A.H.)

**Keywords:** human carbonic anhydrase II, fragment screening, 96-compounds fragment library, Zn^II^ coordination, covalent modification, PanDDA

## Abstract

Fragment screening is a powerful tool to identify and characterize binding pockets in proteins. We herein present the results of a proof-of-concept screening campaign of a versatile 96-entry fragment library from our laboratory against the drug target and model protein human carbonic anhydrase II. The screening revealed a novel chemotype for carbonic anhydrase inhibition, as well as less common non-covalent interaction types and unexpected covalent linkages. Lastly, different runs of the PanDDA tool reveal a practical hint for its application.

## 1. Introduction

Fragments have become an indispensable tool for lead generation in early state drug discovery. The small molecular weight and thereby low molecular complexity, combined with a comparably high degree of functionalization, renders them ideal probes for protein pockets [1,2]. The binding of fragments is usually weak, due to the small number of possible interactions [3,4,5,6]. However, these interactions are generally of high quality, as the low molecular complexity of the fragments enables an optimal orientation within the binding site, and thus facilitates supreme geometrical complementarity between the interacting functional groups of fragment and target, while the risk of deteriorating repulsive interactions of non-optimally placed moieties is reduced [3,4,5,6]. Additionally, they enable, despite the screening of a considerably smaller number of probe molecules, a more efficient coverage of chemical space than drug-like compounds, which increases the possibility of finding a hit in a lead-finding effort with a well-designed fragment library [4,5,6,7]. Detected fragment hits are subsequently elaborated into lead compounds by growing, merging or linking, which relies upon the potentiation of binding affinity upon combination of weak-affinity contributors [3,4,8]. Based on the considerable knowledge about fragment-based research that our group has amassed over the years [9,10,11,12,13,14,15], a 96-entry fragment library was assembled, to make the utility of fragments available to the broader public [16]. We tested its versatility in screenings against several established proteins, among these the well-established drug target and model protein human carbonic anhydrase II (hCAII). The results of this screen are the subject of the present report. 

## 2. Materials and Methods 

### 2.1. Protein Expression and Purification

hCAII was expressed and purified as described previously [17].

### 2.2. Macromolecular Crystallography

Crystals of protein complexes hCAII•1 to hCAII•9 were grown at 18 °C in a mixture of a solution containing 2.70 m ammonium sulfate and 0.1 m TRIS at pH 7.8, which was saturated with para-chloromercuribenzoic acid (PCMB) and protein solution (c = 10 mg mL^−1^) in the final expression buffer (TRIS 0.05 m, pH 7.8). Then, 2 µL of each were mixed on a siliconized cover slip (Jena Bioscience) and placed on the well (24-well plate, Hampton Research), with silicon grease as sealant and 0.5 mL of the first solution in the reservoir [17]. Crystals grew within one day. Crystals were soaked at 18 °C in a solution containing 35% (v/v) aqueous PEG 3350 solution (50% *w*/*v*), 20% (v/v) sodium chloride solution 1.0 m, 25% (v/v) PEG 400, 10% (v/v) water and 10% (v/v) stock solution of the respective fragment in DMSO (1.0 m), for 15 h or 3 min. Crystallographic data were collected on beamlines XRD1 of the ELETTRA synchrotron (Trieste, Italy), P13 operated by EMBL Hamburg at the PETRA III storage ring (DESY, Hamburg, Germany) and 14.1 and 14.2 at the BESSY II electron storage ring, operated by the Helmholtz-Zentrum Berlin, Germany [18,19,20]. Data indexing, integration, and scaling were performed with XDS and XDSAPP2.0 [21,22]. Model building was carried out in Coot [23], with subsequent refinement in Phenix [24]. Crystallographic images were created with PyMOL [25]. Diffraction and refinement data are provided in the Supplementary Materials in Table S1.

### 2.3. Associated Content

#### PDB Accession Codes

**Figure 1:**
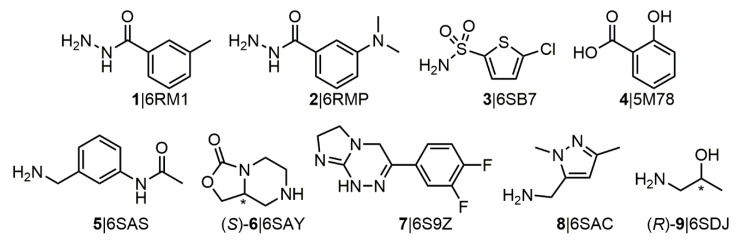
Fragment-sized molecules found in the hCAII crystal structure, comprising library entries **1**–**8** and the putative cryoprotectant **9**. PDB entry codes for the complexes with hCAII are given, as well as the stereochemical information for the bound enantiomers of compounds **6** and **9**; * denotes the stereocenters.

Crystal structures for protein complexes investigated herein will be released upon publication, under the PDB codes shown in Figure 1.

## 3. Results

The structural formulas of fragment-sized ligands that were observed as hits in the crystal structure of hCAII are shown in Figure 1. The complete collection of the 96-entry screening sample can be found in the Supplementary Materials. Molecules **1**–**8** are library entries and 1-aminopropan-2-ol (**9**) was additionally found by chance in the hCAII active site in the course of the search for a new cryoprotectant for hCAII crystals.

**Figure 2:**
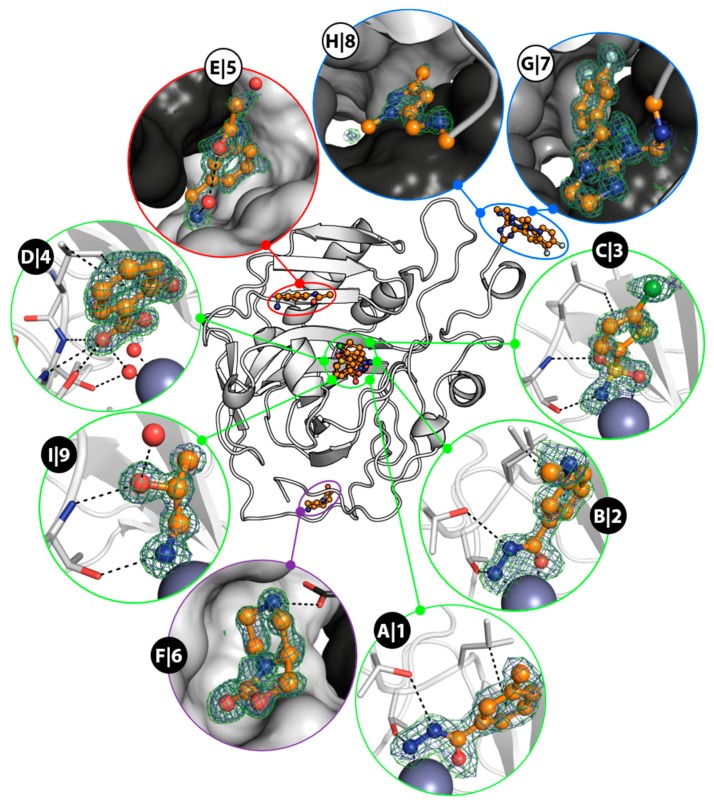
Overview of fragment binding sites. Fragments are shown in orange with omit 2mFo − DFc maps in blue at 1 σ and mFo − DFc maps in green at 3 σ. Active site binders are shown with the interacting amino acids in stick representation. Remotely binding fragments are shown with the surface representation of adjacent protein molecules. Hydrazides **1** (**A**) and **2** (**B**) and sulfonamide **3** (**C**) coordinate to the Zn^II^ cofactor; salicylic acid (**4**, **D**) coordinates to the Zn^II^-bound water molecule. Anilide **5** (**E**) is accommodated in a hydrophobic cavity on the rim of the funnel-shaped active site, which is covered by a proline residue of a symmetry mate and additionally entraps a water molecule. Cyclic carbamate (*S*)-**6** (**F**) coordinates to the *C*-terminus and a Trp sidechain and donates a hydrogen bond to the PCMB molecule of a symmetry mate. Fluorinated compound **7** (**G**) and pyrazole **8** (**H**) are covalently attached to the remainder of the fusion-protein tag from protein expression.

In total, the screening revealed eight hits in the crystal structures of hCAII. Four of these occupy the active site by direct (**1**–**3**) or indirect (**4**) coordination to the Zn^II^ cofactor. These comprise two hydrazides, a chemotype which, to the best of our knowledge, has not been reported for the inhibition of carbonic anhydrases (CAs) so far. Furthermore, one binder was found on the rim of the active site (**5**). Three additional hits were identified in cavities remote from the active site, with **6** mediating a contact between two crystal mates and **7** and **8** unexpectedly covalently linked to the *N*-terminus of the remainder of the fusion-protein expression tag. Aminoalcohol **9** is not an entry of the library referred to herein but was found accidentally during an effort to replace the common cryoprotectant glycerol for other projects [26]. Figure 2 shows an overview of the occupied sites.

## 4. Discussion

### 4.1. Active-Site Binders

**Figure 3:**
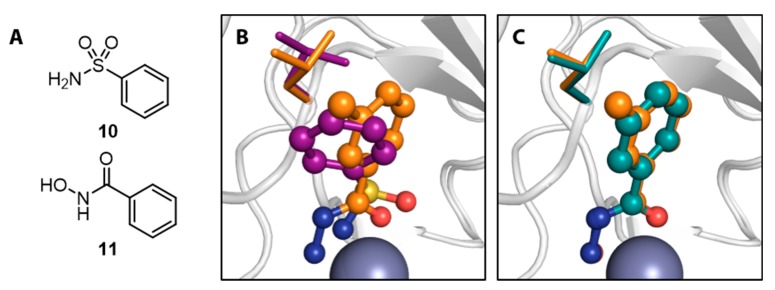
(**A**) Chemical formulas of prototypical hCAII inhibitor benzenesulfonamide (BSA, **10**) and *N*-hydroxybenzamide (**11**). (**B**) Comparison of crystal structures of hydrazide fragment **1** (orange) and BSA (**10**, purple, 6GDC [17]) and the respective side chains of Leu198. (**C**) Comparison of **1** with the analogous *N*-hydroxybenzamide **11** (teal, 4FL7) [27].

Hydrazides **1** and **2** occupy the active site of hCAII and coordinate the Zn^II^ cofactor with the N_β_ and carbonyl O-atom of the hydrazide function, which results in a pentacoordinated cofactor. The second methyl group of fragment **2** was not visible in the electron density. The phenyl rings are involved in a π-interaction with the side chain of Leu198. Notably, the coordination geometry enables a hydrogen bond between N_β_ of the hydrazide group and the side chain hydroxy function of Thr199. This entails a different orientation of the phenyl moiety compared to the complex of hCAII with its prototypical inhibitor benzenesulfonamide **10** (BSA) [17], and triggers a flip of the sidechain of Leu198, which was also observed in the complex of hCAII with *N*-hydroxybenzamide (**11**) by Di Fiore et al. (Figure 3) [27].

Unsurprisingly, sulfonamide **3** can be expected to bind to hCAII, and does so with the conventional geometry. Salicylic acid (**4**) binds in a fashion that was observed previously for other hydroxybenzoic acids in complex with hCAII. Its carboxylate function interacts via a hydrogen bond with the Zn^II^-bound hydroxide ion/water molecule. It furthermore interacts with the side chain of Thr200, a state similar to the pre-binding S-state defined by Gaspari et al. for the binding of BSAs to hCAII and already observed by Martin and Cohen for variously hydroxylated benzoic acid derivatives [28,29]. Surprisingly, aminoalcohol **9**, notably only its (*R*)-enantiomer, is found to occupy the active site. Indeed, sulfonamides are known to bind hCAII as anions, which can be expected to account for the better part of their high affinity for this specific enzyme [30]. Fragment **9** can be assumed to be protonated under the experimental conditions used in the production of the crystal of hCAII-**9** (6SDJ), so the loss of one proton to liberate a lone pair is required before binding. However, it seems unlikely that the primary amine functionality of **9** should lose a second proton to bind as anion. This assumption, combined with the lack of an aromatic π-system which distinctly contributes to the affinity of **10** and its derivatives, suggests a minor affinity of (*R*)-**9** [31]. However, *rac*-**9** was investigated as a putative novel cryoprotectant for hCAII crystals and thus was used in a considerable concentration of 2.6 m, which would compensate for a lack of affinity. Due to its small size and supposedly weak affinity, **9** falls in line with the so-called MiniFrags presented by O’Reilly et al. and impressively demonstrates the capability of weakly binding organic chemical probes to map binding-site properties and to further increase our understanding of protein binding pockets, e.g., with respect to their chiral preferences [32].

### 4.2. Remote Binders

Primary amine **5** was found to occupy a hydrophobic cavity on the rim of the active site funnel (see Figure S1 in the Supplementary Materials). It is held in place by a charge-assisted hydrogen bond with the side chain of Asp72 and furthermore interacts with two water molecules. One mediates a contact between the anilide N-atom and the side chain of Glu69 (W1); the other (W2) is entrapped between the fragment’s primary amine function and the carbonyl oxygen atom of the amide. Additionally, the phenyl ring interacts with the side chain of Ile91 via the π-system, which is furthermore involved in an interaction with the sidechain of Pro237 of a symmetry-related mate. Moreover, an interaction between a lone pair of the carbonyl oxygen atom of the symmetry mate’s amide bond between Pro237 and Glu236 and the fragment’s acetamide group in a fashion similar to the Bürgi–Dunitz trajectory can be inferred [33,34,35]. Thus, the binding of **5** is likely irrelevant for drug discovery, as its binding is enabled by the crystal packing, rather than by genuine affinity for the position on the rim of the hCAII active site.

Fragment **6** was used as racemate. The (*S*)-enantiomer was found to bridge the gap between *C*-terminus and the sidechain of Trp192. Furthermore, the secondary amine is likely protonated and interacts with the carboxylate function of the crystallization agent PCMB of a symmetry-related mate.

### 4.3. Covalently Attached Fragments

**Scheme 1:**
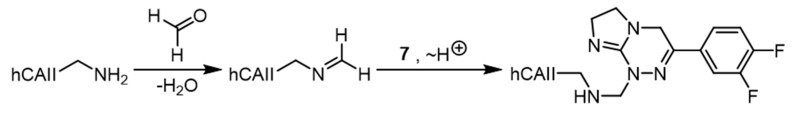
Possible mechanism for the reaction between the *N*-terminus of the hCAII-expression tag protein with 7. The *N*-terminal primary amine reacts with formaldehyde under loss of water to yield an imine. This in turn is nucleophilically attacked by **7** with concurrent proton transfer to yield the final protein-small-molecule hybrid.

Unexpectedly, fragments **7** and **8** were found to be covalently bound to Gly-4, which is part of the remnants of the *N*-terminal fusion protein expression tag. Curiously, **7** is linked to the *N*-terminus, via an unknown atom that is distinctly visible in the electron density. Given the circumstance that the soaking conditions contained a high amount of polyethylene glycol (PEG) 400, the linker can reasonably be assumed to be a methylene unit, which was installed by incorporation of one molecule of formaldehyde, which is known to be present in solutions containing PEG 400 [36]. A possible reaction is shown in Scheme 1.

**Scheme 2:**
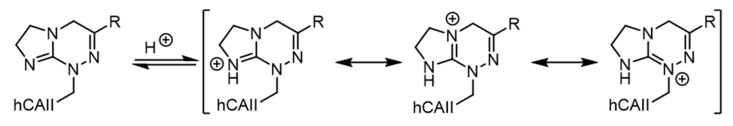
Protonation of the small-molecule moiety of the hCAII–7 hybrid and possible resonance structures. R represents the difluorophenyl substituent.

Fragment **7** itself is involved in some interesting interactions (see Figure S2 in the Supplementary Materials). It is known that the S-atom of a Met side chain is often found in the vicinity of π-donors such as aromatic rings and amide bonds, with which it engages mostly through dispersive interactions [37]. Especially the nucleobase adenine is often found in the company of Met side chains in crystallographic models. While the π -system of adenine is distributed over the whole molecular scaffold, this cannot be the case for compound **7**, given its three CH_2_ units. However, the hydrazinecarboximidamide substructure and the difluorophenyl moiety, which are connected by a C_sp2_ atom, provide an extended π-system that can be expected to act as binding partner for the S-atom of the Met side chain. Given the comparably close distance of 3.3 Å between the O_δ1_ atom of the side chain of Asp71 of a symmetry mate and the covalently linked N-atom of **7**, an interaction can be inferred, which resembles that between an Asp side chain and the positively charged aromatic scaffold of a false-positive fragment screening hit against endothiapepsin, discovered by Cramer et al. [15] It is reasonable to assume that, in fact, a charged interaction is formed, given that the hydrazinecarboximidamide substructure can be considered basic, thus bearing a positive charge that can be distributed over several atoms (Scheme 2), which will furthermore be beneficial for the interaction between **7** and the S-atom of Met1.

**Figure 4:**
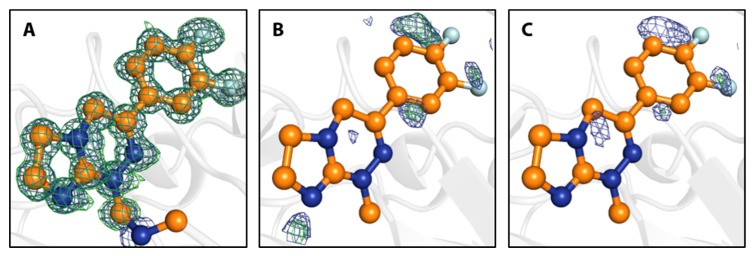
Close-up of the binding site of **7** with electron density maps shown at 1 σ (2mFo-DFc, blue) and 3 σ (mFo-DFc, green). (**A**) Crystal structure of fragment **7** and putative methylene unit as covalent linker to the *N*-terminus, as refined in 6S9Z with omit electron density maps. (**B**) Structure obtained from a crystal soaked in the above trisodium citrate conditions in presence of DMSO. Fragment **7** and the putative methylene unit from 6S9Z, as shown in (**A**), are superimposed. Electron density maps are shown after automated refinement and indicate that **7** does not bind under these conditions. (**C**) Same as (**B**), but for a crystal soaked in the above trisodium citrate conditions in the absence of DMSO.

Furthermore, the side chain of Ser73 of the symmetry mate might interact with the N_3_ atom of **7** that is involved in the delocalization of a putative positive charge. An interaction that is often found for fluorinated compounds is the interaction between an F-atom and an amide bond [38]. The para-F-atom of **7** exerts such an interaction with the amide bond between Gln158 and Lys159 in a symmetry mate, at a distance of 3.2 Å. Furthermore, the distance of 3.2 Å between the meta-F-atom of **7** and the C_δ2_-atom of Leu57 of a different symmetry mate is in the same range as the sum of the van der Waals radii of an aliphatic C-atom and an aromatically bound F-atom of 3.17 Å [39]. Given the unusual observation of a linkage between the protein and ligand, it was tried to reproduce the binding of **7** in a 1.50 m trisodium citrate solution, both with and without the presence of DMSO. The latter salt is a well-known preservative added to prevent decomposition or undesirable chemical changes of pharmaceutical products. Figure 4 clearly shows that **7** is not found in the site described above in either condition. 

**Scheme 3:**
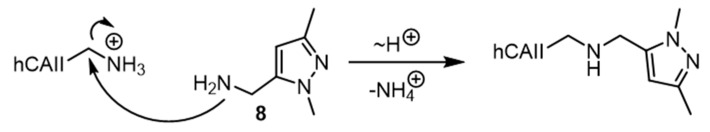
One possible mechanism for the reaction between the *N*-terminus of the remainder of the expression tag of the hCAII-fusion protein with 8. Under the assumption of a protonated *N*-terminal primary amine, the primary amine of compound **8** replaces the *N*-terminus, putatively in an S_N_2 reaction, given the electron withdrawing nature of the adjacent amide bond omitted here.

Fragment **8** is also covalently attached to Gly-4 but without a linker. Rather, a chemical reaction involving only fragment and protein might have occurred through two possible pathways. Either, the primary amine of Gly-4 replaces the protonated primary amine function of **8**, or the reaction proceeds vice versa (Scheme 3).

The presence of both **7** and **8** in this spot can be an indicator for the favorable accommodation of (aromatic) π-systems. Notably, the sidechain of Met1 is not visible in the presence of **8** and might suggest that the π-system of **8** is too small to interact favorably. Furthermore, protonation of the heterocycle is unlikely, given its comparably high acidity (p*K*_a_ = 2.49 at 25 °C [40]), which prevents the formation of a charged interaction with Asp71.

### 4.4. Pan-Dataset Density Analysis

**Figure 5:**
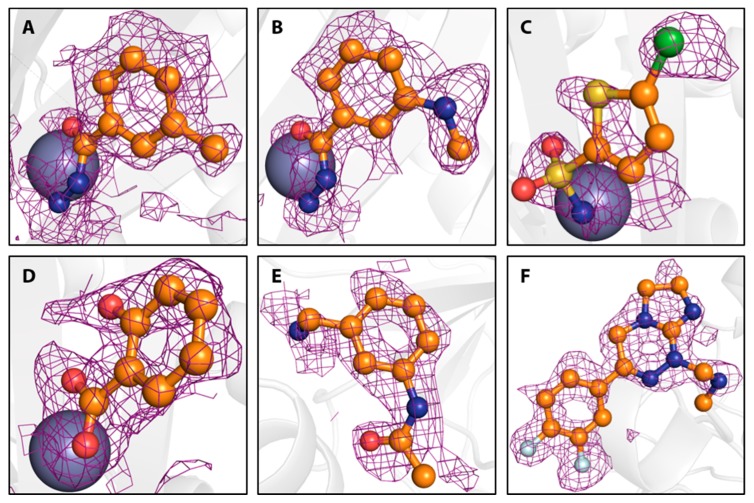
PanDDA density maps for identified fragment binding events shown at 2 σ. (**A**) **1** (**B**) **2** (**C**) **3** (**D**) **4** (**E**) **5** (**F**) **7**.

The weak population of binding sites results in a weak density for the bound ligand, which renders its identification by visual inspection of the electron-density maps firstly speculative, and secondly highly tedious, if not impossible. For a sufficiently large number of crystallographic datasets of the same protein, it is nowadays possible to unveil such binding events by the pan-dataset density analysis (PanDDA) method [41]. PanDDA, in short, relies on a density ‘ground-state’, which is generated as an average of the datasets included in the analysis [41]. A proportion of the ground-state is then subtracted from each individual dataset to reveal ‘changed states’, such as ligand binding [41]. The hCAII datasets collected in the course of the above fragment-screening campaign were subjected to PanDDA. Surprisingly, only fragments **1**, **4** and **5** out of the eight identified hits and no additional binding events were found in this analysis, although visual inspection after a run of the in-house automated refinement pipeline described in reference [12] clearly identified the residual five binders. A putative explanation for the low success rate could have been a non-optimal averaging of datasets to produce the ground-state model. Possibly, this was caused by the fact that soaking times ranged from 15 h at most to three min minimum across the investigated crystals. This became necessary, as we observed that the exposition of crystals to several fragments led to partial or even complete loss of diffraction power, often without visible degradation of the crystals. Such a distinct difference in soaking time can be expected to cause differences, e.g., in the degree to which a binding site that accommodates solvent molecules in the apo-state is occupied by a fragment. Therefore, a PanDDA run was carried out only with datasets from crystals that had been submerged in the soaking drop for 15 h, which resulted in the detection of fragments **1**–**5** and **7**. This analysis still missed fragment **8**, but supports the hypothesis of the influence of deviating soaking times on electron density. However, analysis considering datasets soaked for only 3 min revealed not a single hit but should at least have identified fragment **6**. Figure 5 shows the PanDDA event maps for the identified binding events, based on the 15 h soaking exposure.

## 5. Conclusions

The active site of hCAII is occupied by four out of 96 possible compounds, three of which directly coordinate to the Zn^II^ cofactor. Compounds **1** and **2** are, although not surprisingly, found given the inhibitory action of the structurally highly similar *N*-hydroxybenzamide, to the best of our knowledge the first hydrazide inhibitors of hCAII deposited in the PDB. Although probably not relevant for medicinal chemistry purposes, the binding of four additional fragments in various sites enabled by crystal packing partially revealed that this fragment library is also suited to explore binding sites that feature less common binding partners such as the sulfur atoms of Met and Cys side chains. It has the potential to reveal possibilities for interactions that might not be initially apparent, such as interactions between amide bonds, as for **5**, or the interactions between fluorine atoms and either amide bonds or aliphatic groups. A PanDDA run of all 96 datasets showed a low success rate of only three identified events, out of eight bound fragments that were found by visual inspection of the electron density maps after an automated refinement. A differently assembled PanDDA run, with a subset of datasets obtained from crystals that were all soaked for 15 h, showed an increased number of identified binding events of six bound fragments, and thereby supports the notion, that different soaking times impact the electron density distribution distinctively in crystals. An explanation for this might be that a soaking time of 15 h is sufficiently long to enable full equilibration of all unit cells with ingredients from the surrounding medium. In this context, the unsuccessful recovery of a known binder from a subset of datasets that were exposed to the soaking medium for a drastically shorter period of only three minutes means that the equilibration of the crystal with the soaking drop had not been accomplished. This can be expected to result in distinct differences in the distribution of electron density across unit cells and to be increasingly pronounced with increasing crystal size. This last reasoning would furthermore entail not only drastic differences within one, but across several crystals, and might well be an explanation for the unsuccessful PanDDA run, with datasets obtained after a soaking period of 3 min.

## Supplementary Materials

The following are available online at https://www.mdpi.com/2218-273X/10/4/518/s1, Figure S1: Binding mode of Fragment **5**, Figure S2: Binding mode of Fragment **7**, Figure S3: Binding mode of Fragment **8**, Table S1: Crystallographic data, Table S2: Structural formulas of screened fragments in the 96-entry library; Identified hits are colored in orange and referenced by the numbers used the main article; blue coloring indicates a soaking time of 15 h and green coloring a soaking time of 3 min of the individual fragments.
