# Supplementary material for: A Proof-of-Concept Fragment Screening of a Hit-Validated 96-Compounds Library against Human Carbonic Anhydrase II
Source: Biomolecules. 2020 Mar 29;10(4):518. doi: 10.3390/biom10040518 (PMC7226012; doi:10.3390/biom10040518)
Supplement: Supplementary file 1 [file biomolecules-10-00518-s001.pdf]

Supplementary Materials

A Proof-of-Concept Fragment Screening of a Hit-Validated 96-Compound Library Against Human Carbonic Anhydrase II

Steffen Glöckner, Andreas Heine and Gerhard Klebe

Philipps-Universität Marburg, Marbacher Weg 6, 35037 Marburg, Germany

Table of contents

Table S1	Crystallographic data for hCAII-fragment complexes.	p. 2
Figure S1	Binding mode of Fragment **5**.	p. 4
Figure S2	Binding mode of Fragment **7**.	p. 4
Figure S3	Binding mode of Fragment **8**.	p. 5
Table S2	Formulas of fragments contained in the 96-entry screen	p. 6
References		p. 10

Table S1: Crystallographic data for hCAII in complex with fragments. [a]

	hCAII-**1** (6RM1)	hCAII-**2** (6RMP)	hCAII-**3** (6SB7)	hCAII-**4** (5M78)	hCAII-**5** (6SAS)
**Data collection and processing**					
Beamline	ELETTRA XRD1	ELETTRA XRD1	BESSY II 14.2	DESY P13	BESSY II 14.1
Wavelength / Å	1.0000	1.0000	0.9184	0.7999	0.9184
Space group	$P2_1$	$P2_1$	$P2_1$	$P2_1$	$P2_1$
a,*b*,*c* / Å	42.5, 41.5, 72.0	42.4, 41.6, 72.1	42.5, 41.7, 72.5	42.4, 41.4, 72.2	42.4, 41.5, 72.1
β / °	104.7	104.7	104.3	104.8	104.3
Matthews coefficient / $Å^3$ Da^{-1} [b]	2.1	2.2	2.2	2.2	2.1
Solvent content / % [b]	42.1	44.1	42.9	44.1	42.5
**Diffraction data**					
Resolution range / Å	41.5 – 1.68 (1.78 – 1.68)	41.5 – 1.22 (1.29 – 1.22)	41.7 – 1.09 (1.16 – 1.09)	41.4 – 1.08 (1.083 – 1.08)	41.5 – 1.10 (1.16 – 1.10)
Unique reflections / Å	27053 (4228)	72050 (11150)	98467 (14806)	105910 (16480)	97293 (15236)
$CC_{1/2}$ / %[1]	99.7 (99.2)	99.8 (85.3)	99.8 (93.0)	99.8 (84.6)	99.8 (78.9)
R_{sym} / %[2]	5.1 (7.3)	6.8 (43.3)	5.2 (23.3)	6.6 (42.6)	6.7 (45.8)
Completeness	96.8 (94.5)	98.6 (94.8)	97.2 (91.1)	98.3 (95.2)	97.8 (95.4)
Wilson B factor / $Å^2$	8.7	9.9	8.1	8.9	9.0
Multiplicity	3.8 (3.6)	3.7 (3.7)	3.6 (3.5)	3.7 (3.6)	3.6 (3.6)
I / σ(I)	19.1 (12.1)	10.2 (2.4)	13.5 (4.5)	9.7 (2.2)	9.9 (2.3)
**Refinement**					
Resolution range / Å	35.6 – 1.68	34.9 – 1.22	35.8 – 1.09	35.6 – 1.08	41.1 – 1.10
Reflections used in refinement (work/free)[3] [c]	27052 (25699/1353)	72050 (68447/3603)	98465 (93541/4924)	103053 (97900/5153)	97283 (92418/4865)
Final *R* values for all reflections (work/free)[3][c]	0.153/0.181	0.132/0.162	0.118/0.137	0.125/0.144	0.132/0.149
Protein residues	258	259	258	257	257
Fragment atoms	11	12	10	10	12
Water molecules	166	208	153	152	211
RMSD from ideality					
Bond lengths / Å	0.008	0.007	0.009	0.007	0.006
Bond angles / °	1.00	0.99	1.11	0.97	0.94
Ramachandran plot / % [d]					
Residues in most favored regions	88.0	88.5	88.9	89.9	88.9
Residues in additionally allowed regions	11.5	11.0	11.1	9.6	10.6
Regions in generously allowed regions	0.5	0.5	0	0.5	0.5
Residues in disallowed regions	0	0	0	0	0
Mean *B* factor / $Å^2$ [e]					
Protein non-hydrogen atoms	10.5	12.9	10.8	11.8	11.4
Fragment	8.3	15.5	14.0	17.0	23.5
Water molecules	18.3	24.5	20.7	24.1	22.8

[a] Data in parentheses refer to the highest resolution shell unless stated otherwise. Calculated using the program *Phaser Cell Content Analysis* from the *CCP4* suite [4]. [c] 5 % of all reflections were used for R_{free} calculation. [d] Calculated using the program *PROCHECK* [5]. [e] Calculated using the program MOLEMAN [6].

Table S1 continued. [a]

	hCAII-**6** (6SAY)	hCAII-**7** (6S9Z)	hCAII-**8** (6SAC)	hCAII-**9** (6SDJ)
**Data collection and processing**				
Beamline	BESSY II 14.1	DESY P13	BESSY II 14.1	BESSY II 14.2
Wavelength / Å	0.9184	0.7999	0.9184	0.9184
Space group	$P2_1$	$P2_1$	$P2_1$	$P2_1$
a,*b*,*c* / Å	42.4, 41.5, 72.2	42.4, 41.3, 72.4	42.4, 41.3, 72.3	42.4, 41.1, 72.2
β / °	104.7	104.7	104.8	104.2
Matthews coefficient / $Å^3$ Da^{-1} [b]	2.1	2.1	2.1	2.1
Solvent content / % [b]	42.2	40.2	40.1	39.9
**Diffraction data**				
Resolution range / Å	41.5 – 0.95 (1.01 – 0.95)	41.3 – 0.95 (1.01 – 0.95)	41.0 – 1.02 (1.08 – 1.02)	41.1 – 1.02 (1.02 – 1.08)
Unique reflections / Å	146380 (21821)	141261 (19435)	121056 (19033)	120252 (18157)
$CC_{1/2}$ / %[1]	99.9 (79.5)	99.8 (79.0)	99.8 (78.0)	99.8 (96.0)
R_{sym} / %[2]	5.2 (39.9)	6.3 (43.2)	5.3 (39.1)	4.8 (17.5)
Completeness	96.5 (89.1)	92.7 (79.0)	98.3 (96.0)	98.1 (92.1)
Wilson B factor / $Å^2$	7.9	6.9	8.5	9.6
Multiplicity	3.5 (3.0)	3.8 (3.8)	2.7 (2.5)	3.5 (2.9)
I / $σ(I)$	11.4 (2.0)	10.7 (2.3)	10.0 (2.2)	13.5 (3.7)
**Refinement**				
Resolution range / Å	34.9 – 0.95	35.3 – 0.95	32.0 – 1.02	35.4 – 1.02
Reflections used in refinement (work/free)[3] [c]	146362 (139043(7319)	141257 (134196/7061)	121032 (114983/6049)	120242 (114229/6013)
Final *R* values for all reflections (work/free)[3] [c]	0.122/0.138	0.119/0.137	0.126/0.142	0.128/0.140
Protein residues	264	265	265	258
Fragment atoms	10	17	9	5
Water molecules	223	241	199	180
RMSD from ideality				
Bond lengths / Å	0.007	0.006	0.008	0.006
Bond angles / °	0.97	0.97	1.07	1.01
Ramachandran plot / % [d]				
Residues in most favored regions	89.6	88.3	87.4	88.5
Residues in additionally allowed regions	9.5	11.2	12.1	11.5
Regions in generously allowed regions	0.9	0.4	0.4	0
Residues in disallowed regions	0	0	0	0
Mean *B* factor / $Å^2$ [e]				
Protein non-hydrogen atoms	10.7	8.3	10.7	12.7
Fragment	18.5	6.5	5.8	9.0
Water molecules	20.5	20.4	22.5	24.0

[a] Data in parentheses refer to the highest resolution shell unless stated otherwise. Calculated using the program *Phaser Cell Content Analysis* from the *CCP4* suite [4]. [c] 5 % of all reflections were used for R_{free} calculation. [d] Calculated using the program *PROCHECK* [5]. [e] Calculated using the program MOLEMAN [6].

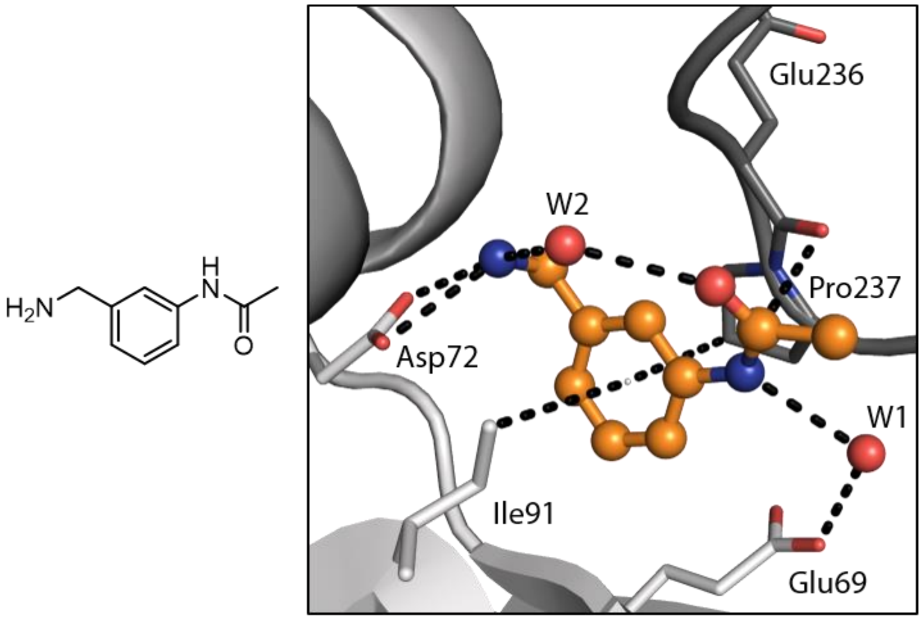

Figure S1: Structural formula and binding mode of fragment **5**. Interactions described in the main article are shown as dashed lines. The phenyl ring centroid is shown as gray dot for clarity. The symmetry-related mate is shown in dark gray.

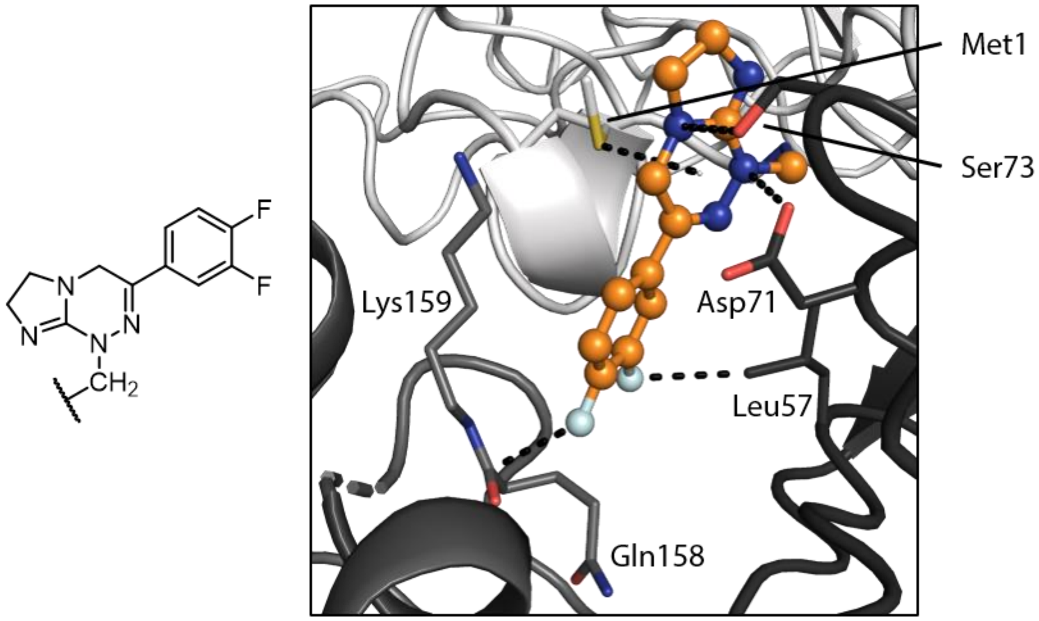

Figure S2: Structural formula and binding mode of fragment **7**. Interactions described in the main article are shown as dashed lines. The centroid of the six-membered heterocycle is shown as gray dot for clarity. Protonation of the heterocyclic scaffold can reasonably be assumed. Symmetry related mates involved in interactions with **7** are shown in darker shades of gray.

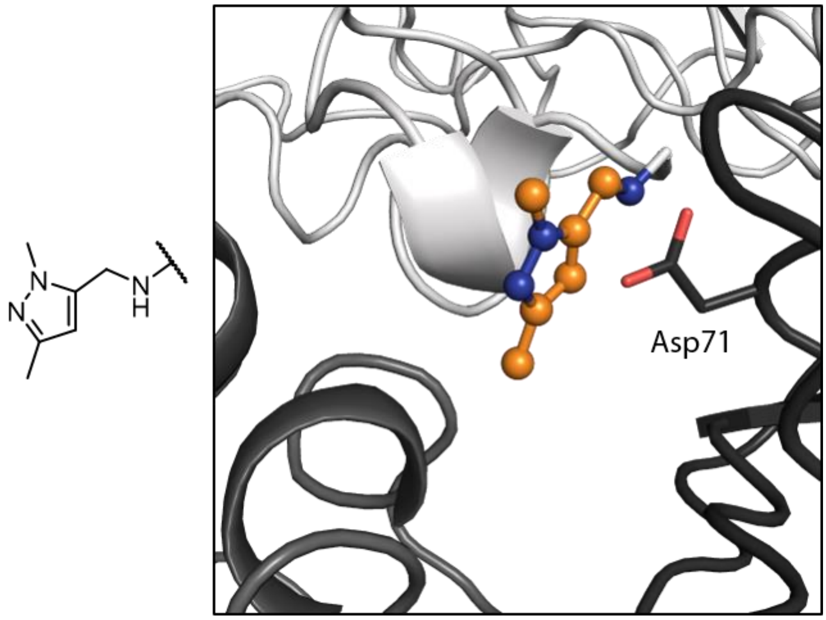

Figure S3: Structural formula and binding mode of fragment **8**. The side chain of Met1 could not be modeled. Protonation of the heterocycle and consequently a charged interaction with Asp71 is unlikely. Symmetry-related mates are shown in darker shades of gray.

Table S2: Structural formulas of screened fragments. Identified hits are colored in orange and the numbers from the main article are given in parentheses. Blue coloring indicates a soaking time of 15 h and green coloring a soaking time of 3 min.

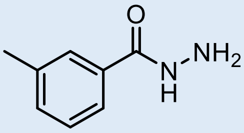	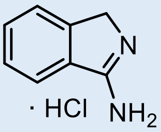	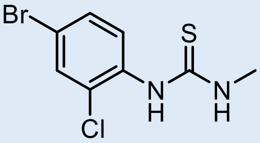
**1** (**1**)	**2**	**3**
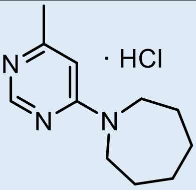	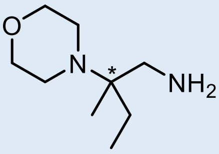	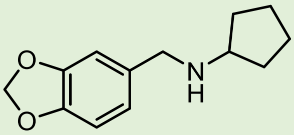
**4**	**5**	**6**
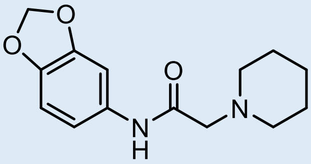	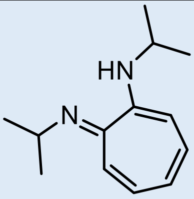	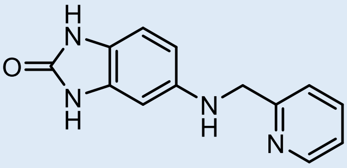
**7**	**8**	**9**[a]
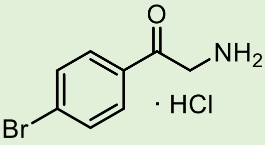	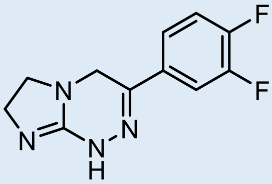	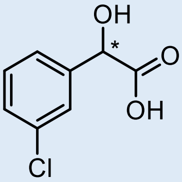
**10**	**11** (**7**)	**12**
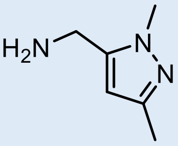	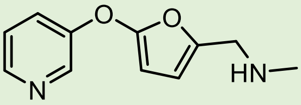	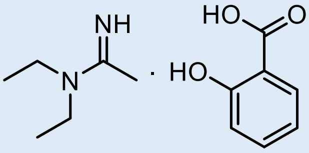
**13** (**8**)	**14**	**15** (**4**)
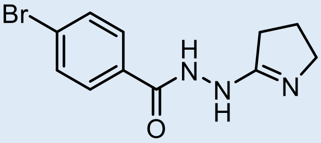	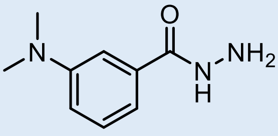	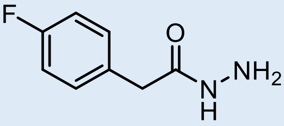
**16**	**17** (**2**)	**18**
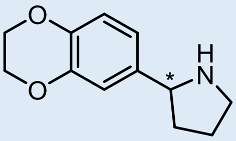	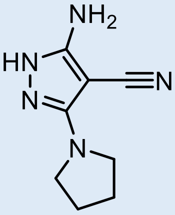	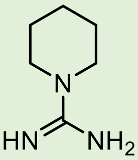
**19**	**20**	**21**
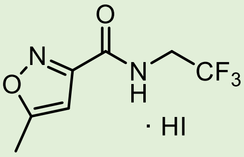	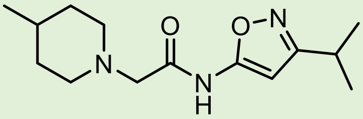	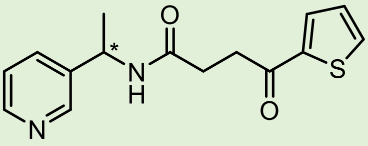
**22**	**23**	**24**

[a] Is *not* fragment **9** from the main article.

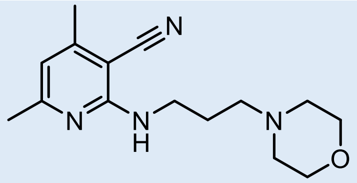	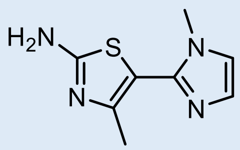	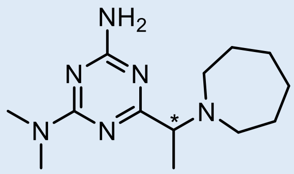
**25**	**26**	**27**
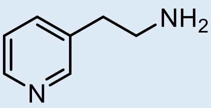	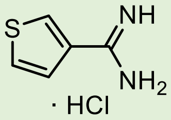	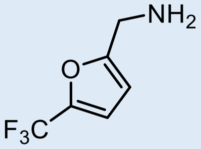
**28**	**29**	**30**
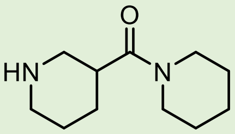	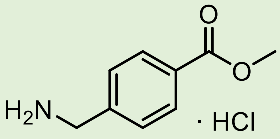	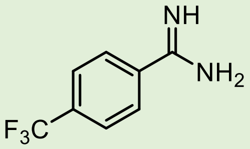
**31**	**32**	**33**
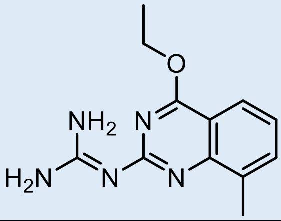	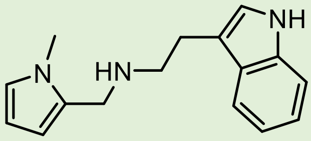	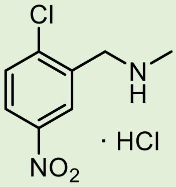
**34**	**35**	**36**
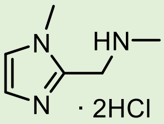	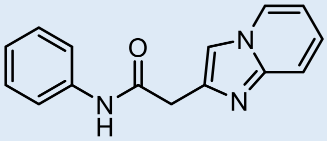	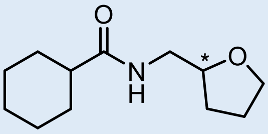
**37**	**38**	**39**
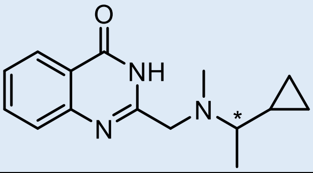	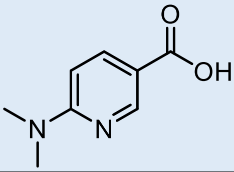	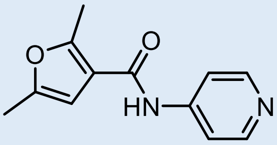
**40**	**41**	**42**
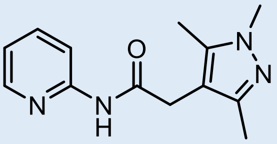	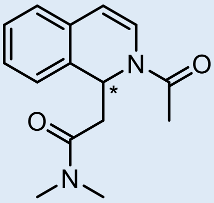	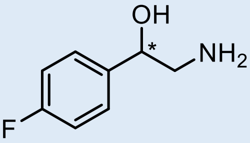
**43**	**44**	**45**
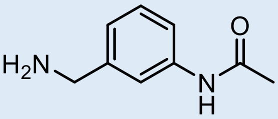	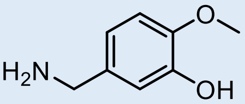	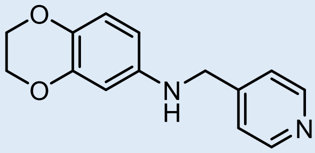
**46** (5)	**47**	**48**

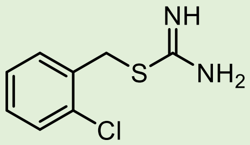	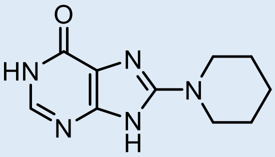	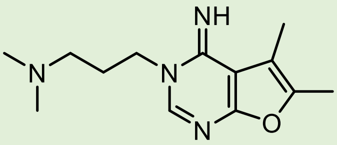
**49**	**50**	**51**
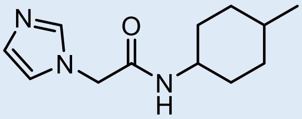	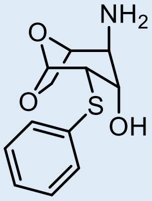	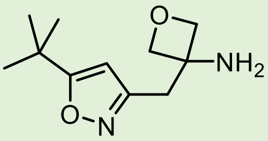
**52**	**53**	**54**
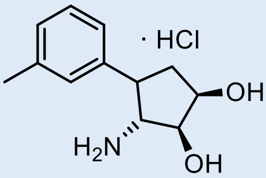	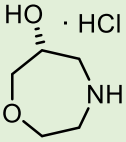	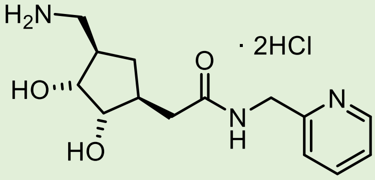
**55**	**56**	**57**
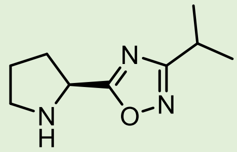	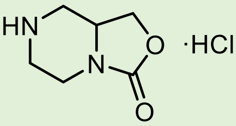	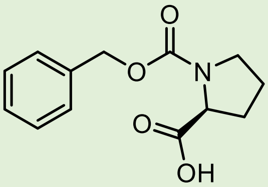
**58**	**59 (6)**	**60**
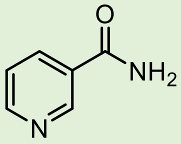	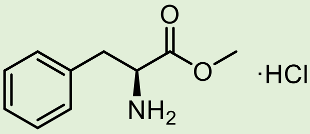	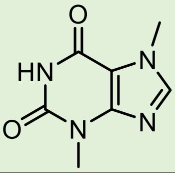
**61**	**62**	**63**
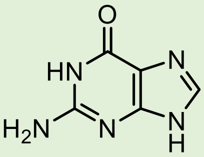	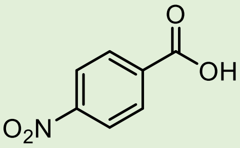	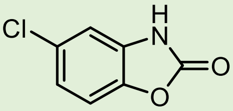
**64**	**65**	**66**
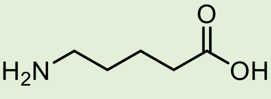	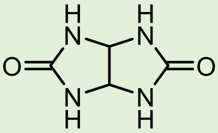	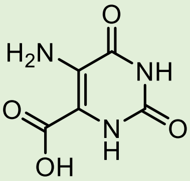
**67**	**68**	**69**
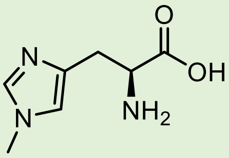	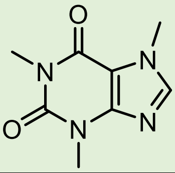	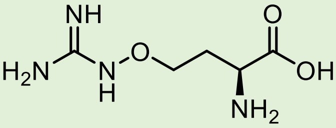
**70**	**71**	**72**

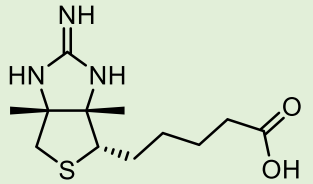	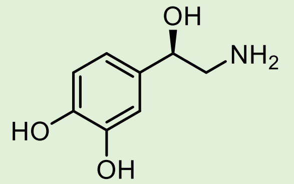	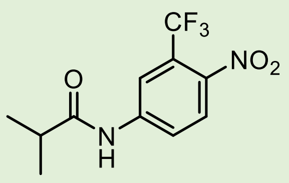
**73**	**74**	**75**
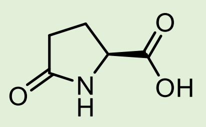	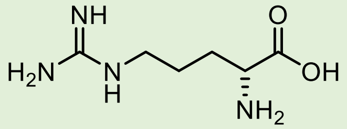	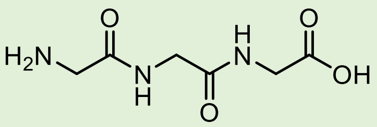
**76**	**77**	**78**
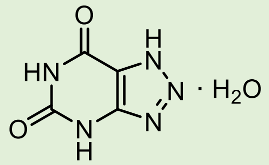	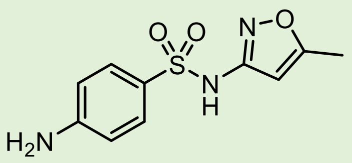	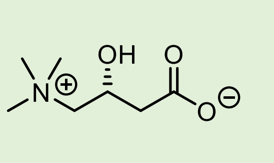
**79**	**80**	**81**
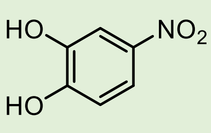	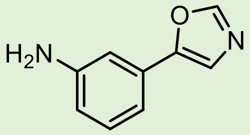	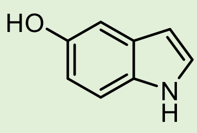
**82**	**83**	**84**
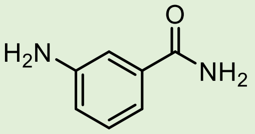	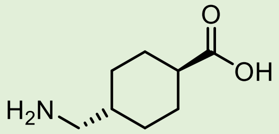	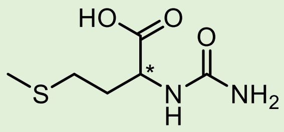
**85**	**86**	**87**
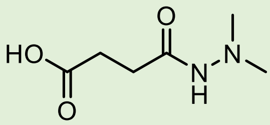	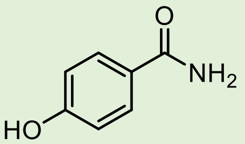	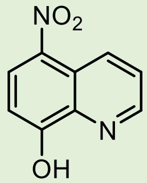
**88**	**89**	**90**
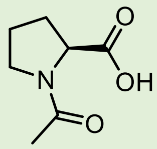	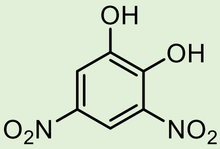	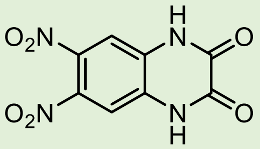
**91**	**92**	**93**
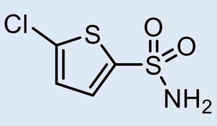	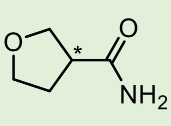	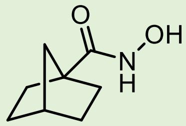
**94** (3)	**95**	**96**

References

1. Karplus, P.A.; Diederichs, K. Linking Crystallographic Model and Data Quality. *Science* **2012**, *336*, 1030–1033.
2. Arndt, U.W.; Crowther, R.A.; Mallett, J.F.W. A computer-linked cathode-ray tube microdensitometer for X-ray crystallography. *J. Phys. E.* **1968**, *1*, 510–516.
3. Brünger, A.T. Free *R* value: a novel quantity for assessing the accuracy of crystal structures. *Nature* **1992**, *355*, 472–475.
4. Winn, M.D.; Ballard, C.C.; Cowtan, K.D.; Dodson, E.J.; Emsley, P.; Evans, P.R.; Keegan, R.M.; Krissinel, E.B.; Leslie, A.G.W.; McCoy, A.; et al. Overview of the CCP4 suite and current developments. *Acta Crystallogr., Sect. D: Biol. Crystallogr.* **2011**, *67*, 235–242.
5. Laskowski, R.A.; MacArthur, M.W.; Moss, D.S.; Thornton, J.M. PROCHECK: a program to check the stereochemical quality of protein structures. *J. Appl. Crystallogr.* **1993**, *26*, 283–291.
6. Kleywegt, G.J.; Zou, J.Y.; Kjeldgaard, M.; Jones, T.A. Around O. In *International Tables for Crystallography, Vol. F. Crystallography of Biological Macromolecules*; Rossmann, M.G., Arnold, E., Eds.; Dordrecht: Kluwer Academic Publisher, The Netherlands, 2001; pp. 353–356, 366–367.
